# Identification of a Novel *FLNC* Truncating Variant in Fetal Tetralogy of Fallot: A Case Report and Review of the Literature

**DOI:** 10.3390/diagnostics15243097

**Published:** 2025-12-05

**Authors:** Zhiqiang Zhang, Dandan Wang, Cong Fang, Linan Xu, Shujing He, Zi Ren, Lei Jia, Xiaoyan Liang

**Affiliations:** 1 Reproductive Medicine Center, The Sixth Affiliated Hospital, Sun Yat-sen University, Guangzhou 510655, China; zhangzhq53@mail.sysu.edu.cn (Z.Z.);; 2 Guangdong Engineering Technology Research Center of Fertility Preservation, Guangzhou 510655, China; 3 Biomedical Innovation Center, The Sixth Affiliated Hospital, Sun Yat-sen University, Guangzhou 510655, China

**Keywords:** *FLNC*, fetus, tetralogy of fallot, cardiomyopathy

## Abstract

**Background and Clinical Significance**: *FLNC* encodes filamin C, a muscle-scaffolding protein crucial for cardiac integrity. Pathogenic *FLNC* variants cause diverse cardiomyopathies (hypertrophic, dilated, restrictive, and arrhythmogenic) and myofibrillar myopathies, but their role in congenital cardiac malformations is unclear. Notably, *FLNC* has not been implicated in structural defects such as Tetralogy of Fallot (TOF) to date. **Case Presentation**: Two fetuses from the same family were prenatally diagnosed with TOF via ultrasound. The trio whole-exome sequencing of the second fetus and her parents identified a novel heterozygous truncating *FLNC* variant (NM_001458.5:c.1453C>T, p.Q485*). Sanger sequencing confirmed the same variant in the earlier TOF fetus. The mother carried the variant but was asymptomatic. *In vitro* mutagenesis in rat cardiomyocytes showed that the mutant *FLNC* construct produced markedly reduced FLNC proteins compared to the wild type and did not form abnormal cytoplasmic aggregates. **Conclusions**: We report on a novel *FLNC* truncating variant associated with fetal TOF, extending the spectrum of *FLNC*-related cardiac anomalies. The variable outcomes among variant carriers—from fetal TOF to adult cardiomyopathy or no clinical manifestations—underscore the complex genotype–phenotype correlations of filaminopathy. This case highlights the importance of comprehensive genetic evaluation in families with diverse cardiac phenotypes and suggests that additional genetic factors likely influence phenotypic expression.

## 1. Introduction

*FLNC* (MIM:102565) encodes filamin C, an actin cross-linking protein abundantly expressed in cardiac and skeletal muscles, and it is critical for maintaining muscle architecture and function [1]. Beyond its structural role, filamin C serves as a scaffold for multiple signaling proteins, mediating diverse cellular pathways that preserve muscle fiber integrity [2]. Variants in *FLNC* are associated with a broad spectrum of cardiomyopathies, including hypertrophic cardiomyopathy (HCM), dilated cardiomyopathy (DCM), arrhythmogenic cardiomyopathy (ACM), restrictive cardiomyopathy (RCM), and isolated distal or myofibrillar myopathy [3]. Notably, *FLNC* variants frequently exhibit variable expressivity and incomplete penetrance [4,5]. Emerging evidence from studies of genomic disorders suggests that such phenotypic variability is often modulated by secondary genetic variants, the polygenic background, and environmental or stochastic factors [6,7]. These findings align with broader observations in neurodevelopmental and cardiac disorders, where multilocus interactions and genetic background contribute substantially to disease expressivity [8,9].

Despite the growing understanding of *FLNC*-associated disorders in pediatric and adult populations, the role of *FLNC* variants in fetal cardiac development remains poorly characterized. Here, we report a novel truncating *FLNC* variant (NM_001458.5:c.1453C>T, p.Q485 *) in a family with two fetuses affected by Tetralogy of Fallot (TOF), a congenital heart defect not previously associated with *FLNC* variants. Family history revealed heterogeneous cardiac phenotypes among carriers, including TOF, highlighting the variable expressivity of this variant and expanding the known clinical spectrum of *FLNC*-related disorders.

This case underscores the importance of comprehensive genetic evaluation in families with diverse cardiac phenotypes and reinforces the notion that variable expressivity in filaminopathies may arise from multilayered genetic and possibly environmental interactions. By documenting this novel association, we aim to broaden the understanding of *FLNC*-related disease mechanisms and their contribution to fetal cardiac anomalies.

## 2. Case Presentation

### 2.1. Pregnancy History and Fetal Findings

**Figure 1:**
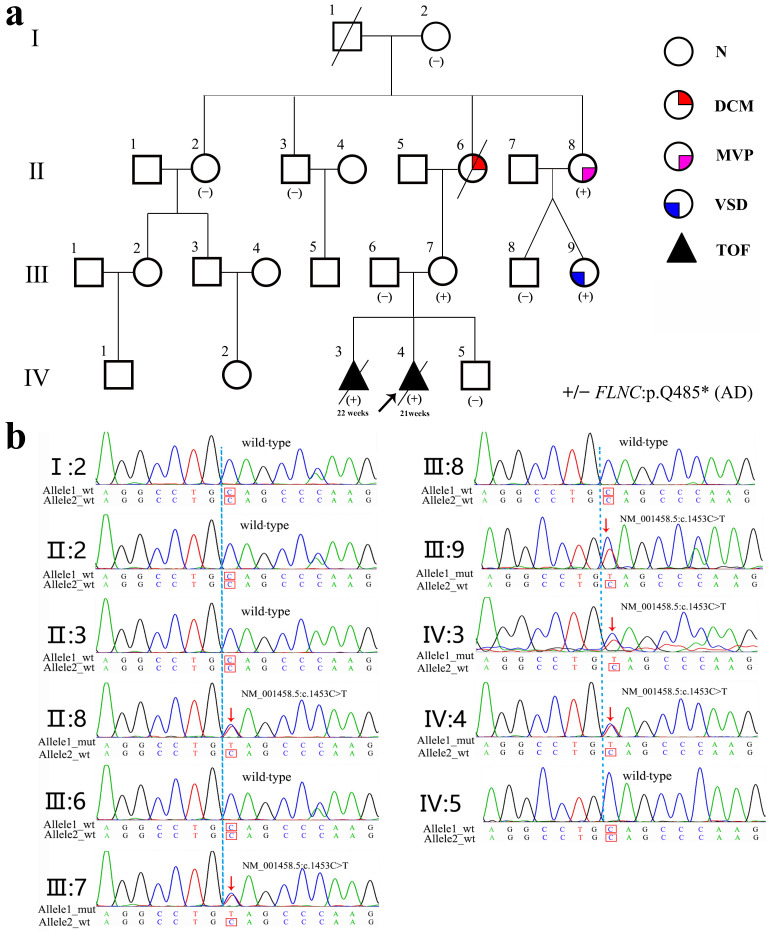
Pedigree of the family affected by an *FLNC* gene variant. (**a**) Pedigree showing a detailed spectrum of cardiac phenotypes. N, DCM, MVP, and ASD denote normal, dilated cardiomyopathy, mitral valve prolapse, and atrial septal defect, respectively. TOF denotes pulmonary valve stenosis, ventricular septal defect, shifting of the aorta, and right ventricular hypertrophy. +/− denotes mutant vs. the wild-type genotype. AD represents autosomal dominant inheritance. In the pedigree, a diagonal slash (/) across a symbol indicates that the individual is deceased. (**b**) Sanger sequence analysis of the *FLNC* gene (NM_001458.5:c.1453C>T, p.Q485*) in members of this family, including affected individuals (II:8, III-7, III-9, IV:3, and IV:4) and wild-type relatives.

**Figure 2:**
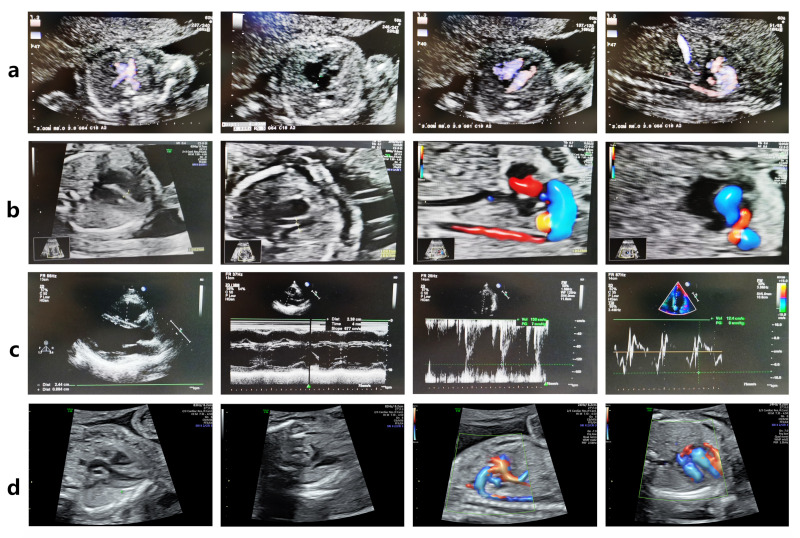
The *FLNC* variant leads to fetal Tetralogy of Fallot (TOF). (**a**) Fetal echocardiography at 22 weeks of gestation revealed TOF (pulmonary valve stenosis, ventricular septal defect, overriding aorta, and right ventricular hypertrophy) in the *FLNC* variant-carrying fetus IV-3. (**b**) Fetal echocardiography at 20 + 3 weeks of gestation revealed TOF in the proband IV-4. (**c**) The woman III-7 carries the *FLNC* variant but has a normal echocardiogram. (**d**) The fetus IV-5, who does not carry the *FLNC* variant, exhibited a normal echocardiogram at 23 weeks of gestation.

In 2017, a 35-year-old woman with a history of infertility (III-7, Figure 1a) conceived through *in vitro* fertilization. Prenatal echocardiography identified TOF in the fetus (IV-3) at 22 weeks of gestation. The diagnosis included pulmonary valve stenosis, ventricular septal defect, shifting of the aorta, and right ventricular hypertrophy, as illustrated in Figure 2a. The karyotype and chromosomal microarray analysis (CMA) results were normal. After genetic counseling and informed consent, the pregnancy was electively terminated.

In 2019, a subsequent embryo transfer resulted in another pregnancy. Fetal echocardiography at 20 weeks and 3 days again revealed TOF in the proband (IV-4) (Figure 2b). The karyotype and CMA results were normal.

### 2.2. Genetic Testing and Variant Confirmation

The trio whole-exome sequencing (trio-WES) of amniotic fluid from the proband and peripheral blood samples from both parents identified a heterozygous nonsense variant in the *FLNC* gene (NM_001458.5:c.1453C>T, p.Q485*) in the proband. This variant, inherited from the mother, was confirmed via Sanger sequencing (Figure 1b). No other pathogenic or likely pathogenic variants associated with congenital heart defects were detected (Table S1).

In 2020, DNA extracted from the formalin-fixed, paraffin-embedded cardiac tissue of the first fetus (IV-3) was analyzed. Due to DNA degradation, WES could not be performed; however, Sanger sequencing confirmed the presence of the same maternal *FLNC* p.Q485* variant.

The variant creates a premature stop codon predicted to result in nonsense-mediated decay (NMD), consistent with a loss-of-function mechanism. The variant is absent from major population databases (1000 Genomes, gnomAD).

### 2.3. Functional Characterization of the FLNC p.Q485* Variant

**Figure 3:**
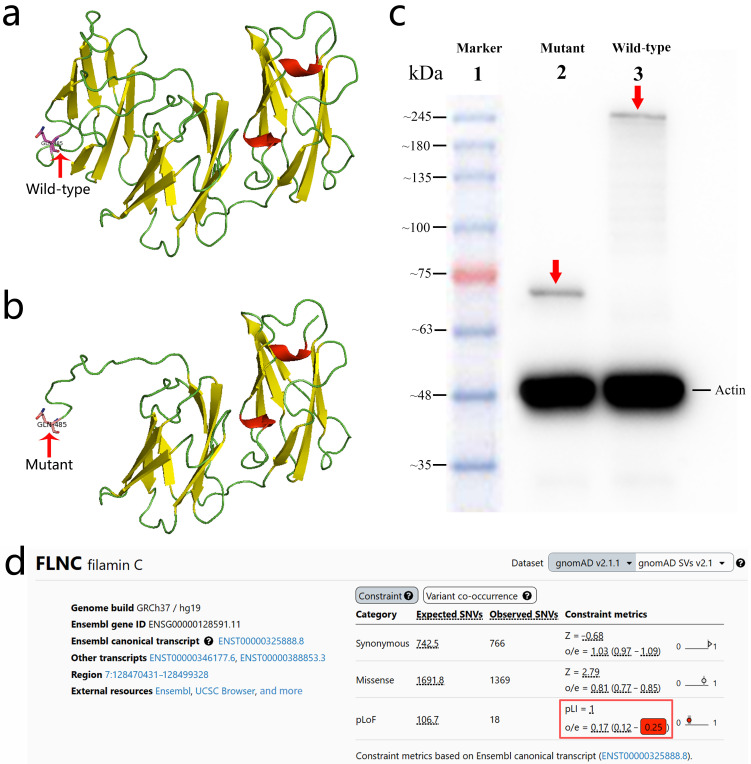
Structural and functional consequences of the *FLNC* (NM_001458.5:c.1453C>T, p.Q485*) variant. (**a**) Predicted 3D structural model of the wild-type FLNC protein. (**b**) Predicted 3D structural model of the mutant FLNC (p.Q485*) protein showing premature truncation and loss of the C-terminal region. (**c**) Western blot analysis demonstrating a truncated ~70 kDa mutant FLNC protein (Lane 2, red arrow) compared with the full-length ~289 kDa wild-type FLNC protein (Lane 3, red arrow). (**d**) gnomAD v2.1.1 constraint metrics for *FLNC*, demonstrating strong intolerance to predicted loss-of-function variants. The gene shows a pLI of 1.0 and an LOEUF of 0.25 (highlighted in the red box), indicating the marked depletion of LoF variants in the general population and supporting a loss-of-function mechanism for the p.Q485* variant.

**Figure 4:**
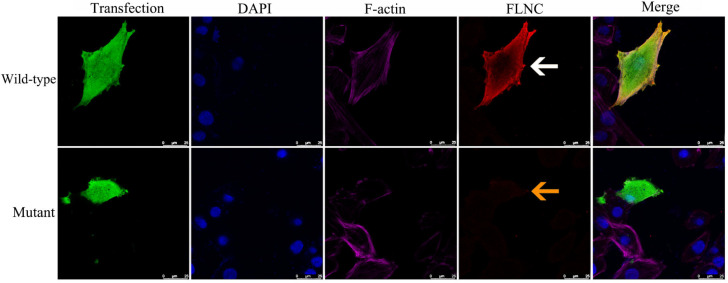
Immunofluorescence analysis of wild-type and mutant FLNC proteins. Immunofluorescence staining was performed in rat cardiomyocyte H9C2 cells transfected with wild-type or truncating *FLNC* constructs. The mutant FLNC protein showed markedly reduced expression (yellow arrow) compared with the wild-type protein (white arrow). All images were captured under identical imaging parameters at 400× magnification. Calibrated scale bars are displayed in the bottom-right corner of each panel to indicate the precise imaging scale. Detailed descriptions of the cell type, plasmid transfection procedures, and antibodies used are provided in the Supplementary Materials.

Structural modeling predicted that the p.Q485* variant results in premature truncation of *FLNC*, resulting in the loss of its C-terminal domains (Figure 3a,b). Functional analyses in a rat-heart-derived cell line demonstrated that wild-type FLNC produced a full-length ~289 kDa protein, whereas the mutant construct yielded a truncated ~70 kDa protein, as shown via Western blotting (Figure 3c). Immunofluorescence staining revealed the markedly reduced expression of the mutant FLNC protein compared with the wild-type control, without evidence of abnormal cytoplasmic aggregate formation (Figure 4).

Together, these findings support a haploinsufficiency mechanism, consistent with the strong loss-of-function constraint of *FLNC*, as reflected by a pLI score of 1.0 and an LOEUF of 0.25 (Figure 3d). According to the ACMG criteria [10], the p.Q485* variant is classified as likely pathogenic (PVS1 + PM2_Supporting).

### 2.4. Family Segregation Analysis and Intrafamilial Variability

First Generation (Great-Grandparents): I-1 (male) died of coronary artery disease at 78; I-2 (female, 90) exhibited no phenotypes and lacked the variant.

Second Generation (Grandparents/Aunts/Uncles): II-2 had no relevant clinical manifestations and did not carry the variant; II-6 had DCM with onset at 30 years and passed away at 57. Although she was not genetically tested, pedigree analyses suggest that she was a carrier of the *FLNC* variant; II-8 had mitral valve prolapse (MVP) and carried the variant.

Third Generation (Mother and Siblings): III-7 (mother) carried the variant but remains asymptomatic (Figure 2c). Among II-8’s children, III-9 carried the variant and was diagnosed with an atrial septal defect (ASD), consistent with previous reports [11], whereas III-8 did not carry the variant and exhibited no cardiac abnormalities.

Fourth Generation (Proband and Sibling): IV-3 and IV-4 both carried the variant and developed TOF.

The pedigree demonstrates an autosomal dominant inheritance pattern, with considerable intrafamilial variability spanning TOF, ASD, MVP, and DCM. The family cardiac phenotype and *FLNC* gene variant status are shown in Table 1.

### 2.5. Preimplantation Genetic Testing (PGT) and Clinical Outcome

In 2022, the family opted for PGT. An embryo confirmed to be negative for the *FLNC* variant was selected for transfer. Prenatal WES and Sanger sequencing at 18 weeks’ gestation verified the absence of the variant, and fetal echocardiography at 23 weeks demonstrated normal cardiac anatomy (Figure 2d). Postnatal echocardiography and electrocardiography were likewise normal.

The child (IV-5)—now 3 years and 2 months old—exhibits normal cardiac structure and function, with appropriate age development and neurodevelopment.

Analyses of the family history revealed notable intrafamilial variability in clinical presentation. The proband IV-4 and individual IV-3 both presented with TOF, whereas the proband’s mother—who carries the same *FLNC* p.Q485* variant—remains asymptomatic. Other affected family members exhibited milder or later-onset cardiac phenotypes, including ASD, MVP, and DCM.

These observations highlight incomplete penetrance and variable expressivity within the family. The pronounced differences between affected and unaffected carriers, including an asymptomatic mother, underscore the importance of comprehensive family screening and individualized clinical management. They also suggest that additional genetic, epigenetic, or environmental modifiers may influence the phenotypic expression of *FLNC* loss-of-function variants.

## 3. Discussion

In recent years, the role of *FLNC* gene variants in cardiomyopathies has received increasing attention. *FLNC* variants are mainly classified into truncating and missense variants, which exhibit distinct clinical phenotypes. Truncating variants typically result in loss of *FLNC* protein function and are associated with cardiac-specific DCM or ACM [12], whereas missense variants are more frequently linked to HCM or RCM [13].

The phenotypic heterogeneity of *FLNC* variants may be closely related to variant type, variant site, and genetic background. For instance, missense variants in the ROD2 domain are associated with characteristic restrictive/hypertrophic phenotypes and a serrated myocardial structure [13]. Moreover, *FLNC* variants may also lead to arrhythmias even in the absence of overt structural abnormalities [14]. Notably, recent studies suggest that phenotypic variability in complex diseases is influenced not only by primary pathogenic variants but also by genetic modifier variants and ascertainment methods [15]. This indicates that individuals carrying the same *FLNC* variant may display markedly different clinical manifestations due to differences in genetic background or detection strategies, providing a new perspective for understanding the variable penetrance of *FLNC* variants.

In the present study, we identified two fetuses carrying the same familial truncating *FLNC* variant, both presenting with TOF, a finding not previously reported in the literature. The mother in this family, who carries the same truncating *FLNC* variant and is currently 41 years old, remains asymptomatic, exemplifying the incomplete penetrance typical of *FLNC* variants [14,16]. This variable penetrance may be partly modulated by genetic modifiers and familial context, supporting Jensen et al.’s concept that the phenotypic expression of complex diseases is influenced by multiple factors [15].

To further identify the genetic cause underlying TOF in the proband, we performed trio-WES and Sanger sequencing on the fetus and its parents. Comprehensive analyses of the coding exons and flanking intronic regions of TOF-related genes reported in OMIM or the literature [17,18,19,20,21,22,23,24] (*NKX2-5*, *GATA4*, *ZFPM2*, *GATA6*, *JAG1*, *TBX1*, *FOXC2*, *TBX5*, *MYH6*, *NOTCH1*, *FLT4*, etc.) revealed no pathogenic or likely pathogenic variants. Among 17 truncating variants detected in different genes, *CFAP57* (NM_152498.3:c.172C>T) and *CCN6* (NM_003880.4:c.624dup) were classified as likely pathogenic and pathogenic, respectively. However, the associated diseases (spermatogenic failure 95 and progressive pseudorheumatoid dysplasia) are unrelated to cardiac abnormalities, and both are inherited in an autosomal recessive manner; the probands were heterozygous carriers. Therefore, their disease relevance and inheritance patterns were inconsistent. In contrast, the *FLNC* (NM_001458.5:c.1453C>T, p.Q485*) variant showed a high concordance with disease association and inheritance pattern and was classified as likely pathogenic (see Table S2).

Western blot analyses of the *FLNC* (NM_001458.5:c.1453C>T, p.Q485*) variant revealed reduced protein levels and a truncated band compared with the wild type (Figure 3c), consistent with haploinsufficiency potentially caused by NMD, as suggested by prior studies of *FLNC* truncating variants [25]. Immunofluorescence analyses also demonstrated that truncating *FLNC* levels were lower than the wild type. This finding is consistent with prior reports suggesting reduced FLNC protein expression (haploinsufficiency) in truncating variant carriers based on immunohistochemistry and molecular studies [12,26]. Importantly, no abnormal cytoplasmic aggregates of the filamentous protein C were detected (Figure 4), supporting a haploinsufficiency model for *FLNC* pathogenesis [12,25]. These observations suggest that reduced *FLNC* expression may impair intercellular adhesion, thereby weakening structural cohesion, which could contribute to cardiac pathology [27].

Previous zebrafish studies have shown that the knockdown or variant of flncb (the zebrafish homolog of human *FLNC*) causes the structural disorganization of muscle fibers and cardiac dysfunction [28] and that the loss of filamin C in zebrafish results in myofibrillar disintegration and protein aggregates in muscle [29]. These findings support a vital role for *FLNC* in muscle and cardiac structural integrity. Additionally, studies indicate that *FLNC* plays a key role in maintaining cytoskeletal integrity and Z-disc structure in cardiomyocytes, and it may also act as a mechanosensor to mediate mechanotransduction [25,27,30,31]. Loss-of-function or actin-binding-deficient variants in Filamin C result in embryonic lethality, ventricular wall rupture, and reduced cardiomyocyte proliferation in mammalian and vertebrate models [32,33,34], underscoring its essential role in myocardial structural integrity.

The cardiac outflow tract (OFT) develops through coordinated contributions from second heart field (SHF) cardiomyocytes and cardiac neural crest cells (cNCCs). SHF progenitors drive outflow tract (OFT) elongation, whereas cardiac neural crest cells (cNCCs) are essential for the septation and alignment of the OFT; perturbations in either lineage can underlie conotruncal defects such as TOF [35,36,37]. Recent zebrafish studies have demonstrated that SHF progenitors directly differentiate into arterial valve cells and contribute to OFT formation [38]—a mechanism highlighted in an expert commentary [39].

Although *FLNC* is mostly studied in adult cardiomyocytes, emerging *in vivo* evidence demonstrates its essential role in embryonic myocardial integrity. The haploinsufficiency of *FLNC* could compromise SHF-derived myocardial structure by disrupting cytoskeletal integrity, Z-disc structure, and mechanotransduction in developing cardiomyocytes, potentially contributing to OFT malformations. Consistently, Cheng et al. suggested that MYOM2 and *FLNC* are associated with both TOF and HCM, supporting the hypothesis that variants in muscle development genes may result in congenital heart defects. Our findings provide a novel perspective linking *FLNC* truncating variants to TOF, highlighting the potential contribution of genetic modifiers and familial context to phenotypic variability [15].

A major limitation of the present study is the inability to perform functional assays using fresh fetal cardiomyocytes. Both fetuses had been terminated before consultation, and only histological myocardial sections were available, preventing primary culture or high-resolution F-actin imaging to directly assess cytoskeletal or Z-disc disruptions.

Future research should incorporate animal models and cellular experiments to investigate the role of *FLNC* variants in cardiac development, particularly their impact on SHF-derived myocardium and OFT formation. Additionally, exploring the influence of genetic modifiers may further elucidate the mechanisms underlying variable penetrance and phenotypic heterogeneity in *FLNC*-related heart defects.

## 4. Conclusions

The present study is the first to report an association between truncating *FLNC* variants and TOF, expanding the clinical and genetic spectrum of filaminopathies. Our findings underscore the critical role of *FLNC* in cardiac development and highlight the variable cardiac phenotypes among family members, reflecting the complexity of the genotype–phenotype relationship. Variants may act in a tissue-specific manner or be modulated by other genetic factors within the family. The present study emphasizes the importance of comprehensive genetic analyses in understanding and managing *FLNC*-related cardiac conditions. Future investigations are warranted to fully elucidate the mechanisms linking *FLNC* variants to cardiac phenotypes, potentially improving diagnostic and therapeutic strategies.

## Figures and Tables

**Table 1:** Family cardiac phenotype and *FLNC* gene variant status.

Generation	Relationship to Proband	Member ID (Figure 1a)	Age/Status	Cardiac Phenotype (Diagnosis and Onset Age)	*FLNC* Variant Status
I	Maternal great-grandfather	I-1	Deceased (78 years)	Coronary artery disease	Unknown
I	Maternal great-grandmother	I-2	90 years	No cardiac abnormalities	No
II	Maternal grandaunt	II-2	70 years	No cardiac abnormalities	No
II	Maternal granduncle	II-3	68 years	No cardiac abnormalities	No
II	Maternal grandmother	II-6	Deceased (57 years)	Dilated cardiomyopathy (onset at 30 years)	Unknown
II	Maternal grandaunt (younger)	II-8	64 years	Mitral valve prolapse (onset at 30 years)	Yes
III	Mother	III-7	41 years	Normal cardiac evaluation	Yes
III	Maternal uncle	III-8	35 years	No cardiac abnormalities	No
III	Maternal aunt	III-9	35 years	Atrial septal defect (onset at 31 years)	Yes
IV	Sister (induced fetus)	IV-3	Terminated pregnancy (24 weeks)	Tetralogy of Fallot	Yes
IV	Proband (induced fetus)	IV-4	Terminated pregnancy (23 weeks)	Tetralogy of Fallot	Yes
IV	Brother	IV-5	3 years 2 months	No cardiac abnormalities	No

Note: Member IDs (e.g., I-1, I-2, II-2, etc.) correspond to the individuals shown in the pedigree in Figure 1a.

## Data Availability

The raw sequence data reported in this study have been deposited in the Genome Sequence Archive (Genomics, Proteomics and Bioinformatics 2021) in the National Genomics Data Center (Nucleic Acids Res 2024); the China National Center for Bioinformation/Beijing Institute of Genomics; and the Chinese Academy of Sciences (GSA-Human: HRA008314), and they are publicly accessible at https://ngdc.cncb.ac.cn/gsa-human.

## Supplementary Materials

The following supporting information can be downloaded at: https://www.mdpi.com/article/10.3390/diagnostics15243097/s1, Table S1: Variants identified by trio whole-exome sequencing in proband IV-4 and carrier status in parents, excluding common polymorphisms; Table S2: Truncating variants identified by trio whole-exome sequencing in proband IV-4 and carrier status in parents, excluding common polymorphisms; Supplementary Methods: Cell culture, Western blot, and immunofluorescence analysis.
