# Supplementary material for: Identification of a Novel FLNC Truncating Variant in Fetal Tetralogy of Fallot: A Case Report and Review of the Literature
Source: Diagnostics (Basel). 2025 Dec 5;15(24):3097. doi: 10.3390/diagnostics15243097 (PMC12731791; doi:10.3390/diagnostics15243097)
Supplement: Supplementary file 1 [file diagnostics-15-03097-s001.zip › Supplementary Materials.pdf]

Supplementary methods

Cell culture and Western blot

The rat cardiac myoblast-derived cell line H9C2 was cultured in Dulbecco's Modified Eagle's Medium (DMEM) supplemented with 10% fetal bovine serum (FBS). Wild-type or mutant FLNC expression vectors were transfected into the cells using FuGENE HD Transfection Reagent (Promega, USA) according to the manufacturer's instructions. Seventy-two hours post-transfection, cells were harvested, and whole-cell lysates were prepared using a Total Protein Extraction Kit (NCM Biotech, China). Proteins were separated by 10% sodium dodecyl sulfate-polyacrylamide gel electrophoresis (SDS–PAGE) and transferred onto polyvinylidene difluoride (PVDF) membranes. Membranes were blocked with 5% nonfat milk in Tris-buffered saline containing 0.1% Tween-20 (TBST) for 2 hours at room temperature. Subsequently, membranes were incubated overnight at 4 °C with primary antibodies against Flag-Tag (1:1000, Sigma-Aldrich) and actin (1:1000, Sigma-Aldrich). After washing, membranes were incubated with HRP-conjugated species-specific secondary antibodies (1:5000, Sigma-Aldrich) for 1 hour at room temperature. Protein bands were visualized using an Electro-Chemi-Luminescence (ECL) reagent (Merck Millipore, Germany).

Immunofluorescence analysis

Following transfection, cells were fixed in 4% paraformaldehyde, permeabilized with 0.5% Triton X-100, and blocked with 5% bovine serum albumin (BSA) for 1 hour at room temperature. Cells were then incubated overnight at 4 °C with primary antibodies against Flag-Tag (1:200, Sigma-Aldrich) diluted in 5% BSA. After washing, cells were incubated with a Fluor647-conjugated goat anti-mouse IgG secondary antibody (1:500, Affinity) for 1 hour at room temperature. F-actin was labeled with Phalloidin-iFluor 555 (AAT Bioquest, USA) for 1 hour at room temperature, and nuclei were stained with Hoechst 33342 (Meilunbio, China). Fluorescent images were acquired using a Leica laser scanning confocal microscope.
